# Evaluating the association of *TRPA1* gene polymorphisms with pain sensitivity: a protocol for an adaptive recall by genotype study

**DOI:** 10.1186/s12920-022-01156-5

**Published:** 2022-01-12

**Authors:** Aidan P. Nickerson, Laura J. Corbin, Nicholas J. Timpson, Keith Phillips, Anthony E. Pickering, James P. Dunham

**Affiliations:** 1 School of Physiology, Pharmacology and Neuroscience, University of Bristol, Bristol, BS8 1TD UK; 2 Anaesthesia, Pain and Critical Care Sciences, University of Bristol, Bristol, UK; 3 Eli Lilly and Company, 8 Arlington Square West, Bracknell, RG12 1WA UK; 4 MRC Integrative Epidemiology Unit, University of Bristol, Bristol, BS8 2BN UK; 5 Population Health Sciences, Bristol Medical School, University of Bristol, Bristol, BS8 2BN UK

**Keywords:** ALSPAC, Recall by genotype, Adaptive design, Pain, Quantitative sensory testing, TRPA1

## Abstract

**Background:**

Pain is a complex polygenic trait whose common genetic underpinnings are relatively ill-defined due in part to challenges in measuring pain as a phenotype. Pain sensitivity can be quantified, but this is difficult to perform at the scale required for genome wide association studies (GWAS). Existing GWAS of pain have identified surprisingly few loci involved in nociceptor function which contrasts strongly with rare monogenic pain states. This suggests a lack of resolution with current techniques. We propose an adaptive methodology within a recall-by-genotype (RbG) framework using detailed phenotyping to screen minor alleles in a candidate ‘nociceptor’ gene in an attempt to estimate their genetic contribution to pain.

**Methods/design:**

Participants of the Avon Longitudinal Study of Parents and Children will be recalled on the basis of genotype at five common non-synonomous SNPs in the ‘nociceptor’ gene transient receptor potential ankylin 1 (*TRPA1*). Those homozygous for the common alleles at each of the five SNPs will represent a control group. Individuals homozygous for the minor alleles will then be recruited in a series of three sequential test groups. The outcome of a pre-planned early assessment (interim) of the current test group will determine whether to continue recruitment or switch to the next test group. Pain sensitivity will be assessed using quantitative sensory testing (QST) before and after topical application of 10% cinnamaldehyde (a TRPA1 agonist).

**Discussion:**

The design of this adaptive RbG study offers efficiency in the assessment of associations between genetic variation at *TRPA1* and detailed pain phenotypes. The possibility to change the test group in response to preliminary data increases the likelihood to observe smaller effect sizes relative to a conventional multi-armed design, as well as reducing futile testing of participants where an effect is unlikely to be observed. This specific adaptive RbG design aims to uncover the influence of common *TRPA1* variants on pain sensation but can be applied to any hypothesis-led genotype study where costly and time intensive investigation is required and / or where there is large uncertainty around the expected effect size.

*Trial registration*: ISRCTN, ISRCTN16294731. Retrospectively registered 25th November 2021.

**Supplementary Information:**

The online version contains supplementary material available at 10.1186/s12920-022-01156-5.

## Background

Pain is a cognitive motivational state whose function is to minimise the risk of injury and to aid healing and recovery. There is a large variation across the population in pain experience as well as apparent susceptibility and twin studies have suggested that the genetic heritability may be moderate (35–50%) [1]. There are a number of examples of rare, highly penetrant single nucleotide polymorphism (SNPs) modulating pain sensitivity, including in transient receptor potential ankyrin 1 (*TRPA1*) [2, 3]. However, the genetic contribution to most acute and persistent pain is likely comprised of the cumulative effect of many SNPs with small effects [4].

The most common approach to understanding the association of individual SNPs in polygenic traits is to perform a genome wide association study (GWAS). However GWAS have more power if there is a well-defined, relatively homogeneous phenotype with which to search for genetic associations across individuals. The more heterogeneous the phenotype, the lower the probability of identifying meaningful associations. Pain is a complex biological, psychological and social phenomenon [5] where multiple pain mechanisms can be in play to differing extents at any one time. This results in an intrinsically heterogeneous phenotype even within clinically defined patient populations. This heterogeneity within pain phenotypes then requires a very large cohort for SNP effects to be observed in a GWAS which, due to practicality, limits the assessment of individuals to phenotyping tools that are often questionnaire-based, reliant on recall and therefore lack mechanistic specificity and are subject to report bias. A recent large-scale GWAS of multisite chronic pain conducted in the UK Biobank identified 76 independent genome-wide significant SNPs and estimated SNP heritability to be 10% [6]. This GWAS revealed similarities in the genetic profile of pain to common comorbid mental health conditions like major depressive disorder and generalised anxiety disorder. However, none of the associated SNPs were specific to the pain transduction pathway (including *TRPA1*) likely due to a lack of mechanistic sensitivity of the questionnaire approach.

Quantitative sensory testing (QST) uses controlled and reproducible stimuli to evoke a percept, which is measured using standardized language and pain scales. This approach enables quantification of an individual’s pain perception with more mechanistic precision than simple pain scores. The German Research Network on Neuropathic Pain (DFNS) have produced a comprehensive protocol and corresponding reference values which is an accepted standard in the field [7]. This protocol has been used to identify defined patient sub-populations and predict efficacy of drugs [8, 9]. Unfortunately, the cost and time required to test the number of participants required to perform a GWAS of pain sensitivity using QST makes such an approach challenging, although a study design has recently been proposed to test 1500–2000 healthy young subjects [10].

Where there is a strong hypothesis for a candidate gene to alter function, informed by knowledge of biological mechanisms, a recall-by-genotype (RbG) study can be used. In this design, individuals with known variations in candidate genes are recalled for targeted detailed phenotyping. This selective recruitment reduces genetic variability of the cohort within genes expected to be involved in the trait of interest and allows more robust phenotyping with lower measurement error, therefore increasing the power of the study to detect a difference (as compared to random sampling from the population), allowing the study to be conducted on a smaller cohort than would otherwise be required. Given random allocation of alleles at conception, the RbG design generates study groups in which confounding factors are on average equal enabling a potentially informative assessment of genotypic association [11]. There is a wealth of evidence from studies of fundamental pain neurobiology that can be used to inform mechanistic candidate gene investigations [12], such as the gene families of transducer proteins involved in sensing threatening stimuli.

We have chosen to focus on one candidate gene of interest in acute and chronic pain, *TRPA1.* This nociceptor transducer protein is a cation channel that is activated by thermal, mechanical and chemical stimuli including mustard oil and cinnamaldehyde [13]. Additionally, *TRPA1* is upregulated in response to inflammation [3] which is a precursor to chronic pain [14]. TRPA1 also plays a pivotal role in reactive airway diseases such as asthma [15, 16]. Based on data in dbSNP [17], we selected common *TRPA1* SNPs (minor allele frequency (MAF) > 1%) which represent nonsynonymous mutations (i.e. involve an amino acid change within the TRPA1 protein). A review of the literature has suggested that these SNPs associate with altered channel function (see Table 1). Given the MAF range of the selected SNPs, the implementation of a RbG study in the Avon Longitudinal Study of Parents and Children (ALSPAC, genetic data on ~ 8000 young adult participants) [18] represents a feasible and efficient study design.

**Table 1:** *TRPA1* SNP information

*TRPA1* allele group #	SNP (Rs ID)	Major (control) allele^a^	Minor allele	MAF in ALSPAC^b^	MAF in 1000G^c^	Amino acid position (number and domain) with amino acid change relative to reference	Functional evidence
1	rs7819749	G	T*	0.40	0.39	K186N (ANK4)	K186 (resulting from the minor allele in ALSPAC), has increased response to CFA relative to N186, with similar responses to other agonists [19]
2	rs920829	C*	T	0.10	0.13	E179K (ANK4)	Patients with paradoxical heat sensations show a lower frequency of being either hetero- or homozygous for the minor allele [20] E179, shows cold evoked calcium flux whereas K179 does not [21] K179 has reduced response to CFA relative to E179 [19].^#^ Individuals hetero- and homo-zygous for the minor allele have increased odds of asthma [15] Patients with the minor allele have more presentations to healthcare with sickle cell pain [22]
	rs959976	T*	C	0.16	0.20	H1018R (cytoplasmic)	The presence of the minor allele increases the odds of doctor diagnosed asthma [15] R1018 had increased response to coal fly ash relative to H1018 [19].^#^
3	rs16937976	C*	G	0.15	0.17	R58T (cytoplasmic)	C3 and T58 separately, but not when co-expressed, have increased response to CFA, AITC and DTBP relative to R58/R3 [19].^#^
	rs13268757	G*	A	0.15	0.17	R3C (cytoplasmic)	

*Indicates the reference allele in dbSNP – note that this is different to the predicted ancestral allele for rs7819749. SNP information is extracted from dbSNP and is therefore reported in the forward orientation whilst *TRPA1* itself maps to the reverse strand. dbSNP: build 154, GRCh38, last accessed: 27th January 2021) [17]. SNPs in high linkage disequilibrium as reported in LDLink using GBR cohort [23]: Group 2 LD: r^2^ = 0.51. Group 3 LD: r^2^ = 1. AITC: Allyl isothiocyanate, ALSPAC: Avon Longitudinal Study of Parents and Children, CFA: Coal fly Ash, DTBP: 3,5-*Ditert*-butylphenol, MAF: minor allele frequency, SNP: single nucleotide polymorphism

^#^Deering Rice et al., 2015 expressed TRPA1 with site directed mutations HEK cells; “response” relates to calcium flux evoked by the stated TRPA1 agonists

^a^In all cases the major allele is also the designated ancestral allele in dbSNP

^b^MAF is as reported in dbSNP

^c^MAF in 1000 Genomes Project phase3 release V3+

Adaptive study designs are commonly used in clinical trials to optimise the number of participants recruited to trial arms (for a review see [24]). Adaptive trial designs are commended within the Initiative on Methods, Measurement, and Pain Assessment in Clinical Trials (IMMPACT) guidelines [25]. A typical adaptive design utilises interim analysis performed by an independent data monitoring committee after a pre-specified number of individuals are recruited. The committee can be unblinded to the treatment group allocation to enable evaluation of the probability of success—either futility or efficacy, balanced against any associated toxicity findings. At interim assessment, the committee can choose to adapt the study which can mean: study termination, sample size adjustments, altered recruitment strategy or even change to the primary endpoints (for a review see Pallmann, Bedding [26], Bauer and Brannath [27]).

When applied in the context of a RbG study, an adaptive design should increase the likelihood of observing smaller SNP effects on phenotype, prevent unnecessary testing in the case of futility and enable screening of multiple alleles by altering the recruitment strategy early if futility is demonstrated. Due to its prevalence in clinical drug trials the statistical implications of interim assessment have been well studied [28]. To the best of our knowledge, the proposed adaptive RbG study design is a novel methodology that offers a number of advantages and is potentially generalisable to many other settings and study questions.

## Methods/design

This study aims to investigate the association of common variants of *TRPA1* with altered pain sensitivity within the Avon Longitudinal Study of Parents and Children (ALSPAC) cohort who are a regionally representative cross-sectional population aged around 30 years (with a correspondingly relatively low incidence of chronic pain). Five *TRPA1* SNPs known to introduce missense mutations and with minor allele frequencies of > 1% hypothesized to impact TRPA1 function (see Table 1) will be investigated. The effect of these five SNPs will be assessed in three groups due to the high linkage disequilibrium between two pairs of minor alleles. QST results from the individuals in these three test groups will be compared to those of a reference group who are homozygous for the major (ancestral) allele at all five SNPs. The results will be subject to planned interim assessments for futility to alter recruitment if there is low probability of success of detecting a phenotype for a given allele until a maximum of 100 participants have been assessed.

Heat pain threshold is the primary outcome in this study as in both healthy volunteers and animal models TRPA1 is involved in determining heat pain sensitivity, particularly in the sensitised state [29–32].

### Ethical considerations and informed consent

The study was presented to the ALSPAC Original Cohort Advisory Panel (OCAP). Ethical approval for the original study was obtained from the ALSPAC Ethics and Law Committee and the Local Research Ethics Committees. Informed consent for the use of data collected via questionnaires and clinics was obtained from participants following the recommendations of the ALSPAC Ethics and Law Committee at the time. The proposal number was B3236 and the approval number is 94082. All subjects will provide written informed consent and will be reimbursed for their time and travel costs. This study is sponsored by the University of Bristol.

### Participant recruitment

ALSPAC is a transgenerational prospective birth cohort that began with the recruitment of 14,541 pregnant women resident in Avon, UK with expected dates of delivery 1st April 1991 to 31st December 1992. Since then, the health and development of mothers and their children has been followed across the life-course. When the oldest children were approximately 7 years of age, an attempt was made to bolster the initial sample with eligible cases who had failed to join the study originally. As a result, the total sample size for analyses using any data collected after the age of seven is therefore 15,454 pregnancies, resulting in 15,589 foetuses. Of these 14,901 were alive at 1 year of age [18, 33, 34]. Please note that the study website contains details of all the data that is available through a fully searchable data dictionary and variable search tool: http://www.bristol.ac.uk/alspac/researchers/our-data/.

For this study, members of the original ALSPAC cohort (individuals born between 1990 and 1992) will be selected for an invite based on their genotype at the five SNPs using previously acquired genetic data, please see Additional file 1, ALSPAC: Genotyping and imputation description. Individuals with the required genotypes will be identified using genome-wide data, as imputed to the Haplotype Reference Consortium (v1.1) reference panel [35]. Only individuals whose genotypes at the five SNPs were imputed with a probability of > 0.99 were eligible for invite. The SNPs of interest were rs7819749, rs959976 with rs920829, rs16937976 with rs13268757 (dbSNP build 154, GRCh38.p12 [17]) and a control group of individuals homozygous for all five major alleles (see Table 1 for details). Investigators and participants will remain blind to genotype throughout the recruitment and data collection phases of the study.

Invitations will be sent to selected ALSPAC participants, together with a participant information sheet and reply slip. All participants who volunteer to take part will undergo telephone screening with exclusions applied based on the following criteria:

- Neurological disorders including peripheral neuropathy
- Regular use of analgesics
- Any pain medication taken within 24 h of QST
- Pregnancy
- Acute or chronic pain conditions
- Severe anxiety/depression
- Allergy to cinnamon, mustard, alcohol/chlorhexidine wipes, latex.
- Use of non-prescribed or recreational drugs (assessed by questionnaire).

## Data collection

### Quantitative sensory testing

**Fig. 1:**
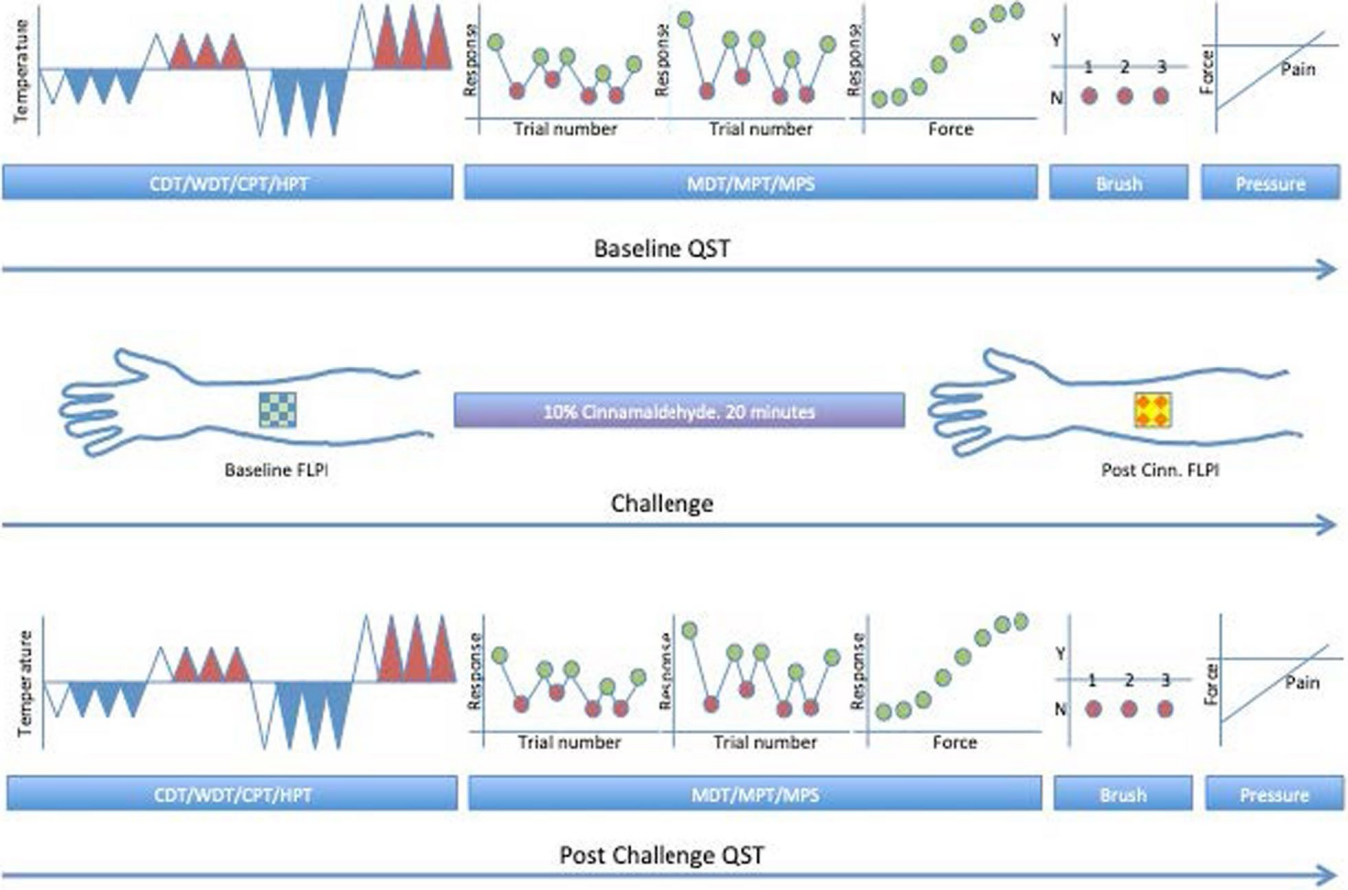
Schematic of Quantitative Sensory Testing (QST) protocol. Top row represents the baseline QST including thermal and mechanical stimulation. The middle row shows capture of baseline cutaneous perfusion using the FLPI in the 4 × 4 cm region of interest, application of 10% cinnamaldehyde and then capture of the post challenge cutaneous perfusion. The bottom row represents the post challenge QST. (Figure adapted from Rolke et al. [7]). CDT, cold detection threshold; WDT, warm detection threshold; CPT, cold pain threshold; HPT; heat pain threshold; MDT, mechanical detection threshold; MPT, mechanical pain threshold; MPS; mechanical pain sensitivity; Brush, presence or absence of brush allodynia; Pressure, deep pressure pain threshold; FLPI, full field laser perfusion imaging; Cinn, cinnamaldehyde

The participants will be assessed using quantitative sensory testing (QST, see protocol in Fig. 1) before and after sensitisation by topical application of 10% cinnamaldehyde (a known activator of TRPA1). QST paradigms are based upon the DFNS protocol [7], streamlined in line with the primary hypothesis to omit some non-nociceptive assessments.

Thresholds for heat and cold detection and pain will be tested using a thermode (Medoc TSA-II, Medoc, Israel, or similar) on the right volar forearm. The temperature of the thermode will change at 1 °C per second until the participant reports either detection of temperature change (cool and warm detection threshold), or detection of pain (heat or cold pain threshold) via a mouse click. The thermode then returns to a neutral temperature of 32 °C. The first trial will be discarded as an acclimatisation and then followed by 3 experimental repeats.

Thresholds for innocuous mechanical stimuli will be assessed using calibrated von Frey filaments (TouchTest; Stoelting, USA) via the method of levels. Mechanical pain thresholds, again via the method of limits, and stimulus response curves will be assessed using calibrated punctate needle stimulators (PinPricks; MRC Systems, Germany). For the stimulus response curve participant numerical pain ratings from 0 (no pain) to 100 (worst imaginable pain), will be assessed 5 times with 7 filaments exerting forces from 8 to 512 mN presented in a randomised manner. Dynamic mechanical allodynia will be assessed with 5 standardised brush strokes (SenseLab; via MRC Systems, Germany). Pressure pain sensitivity will be assessed with an algometer (Somedic, Sweden) applied over the muscles of the right volar forearm. Skin perfusion imaging,  axonal flare etc. Axonal flare in response to cinnamaldehyde will be measured using full-field laser perfusion imaging (FLPI) of the target area of skin before and 20 min after sensitization [36] (moorFLPI-2; Moor Instruments). Full field laser perfusion imaging (also known as laser doppler perfusion imaging) quantifies skin perfusion by detecting alterations in reflected laser light resulting from the movement of blood under the skin. Activation of nociceptors in the skin produces a local flare because of release of vasoactive substances which causes an increase in perfusion. This method has previously been used to study the effects of TRPA1 activation by agonists such as cinnamaldehyde [32] and also as a secondary end point in studies of TRPV1 antagonists [37].

#### Cinnamaldehyde application

After baseline QST and skin perfusion imaging the skin will be sensitized by application of trans-cinnamaldehyde (Sigma Aldrich) 10% in ethanol (1 ml) to a 4 × 4 cm area of the participant’s volar forearm for 20 min using a dressing pad covered with an occlusive adhesive dressing. This concentration of cinnamaldehyde (10%) is the lowest concentration known to reliably activate nociceptors and elicit pain and flare. Lower concentrations predominantly evoke itch [38]. The 20 min duration is informed by prior publications measuring thermal thresholds and flare [29, 32, 39]. Participants will then be asked to rate the cinnamaldehyde evoked pain from 0 (no pain) to 10 (worst pain imaginable). They will then be asked to described any evoked sensations. The FLPI and QST will then be repeated.

Study data will be collected and managed using REDCap electronic data capture tools hosted at the University of Bristol [40, 41]. REDCap (Research Electronic Data Capture) is a secure, web-based software platform designed to support data capture for research studies, providing (1) an intuitive interface for validated data capture; (2) audit trails for tracking data manipulation and export procedures; (3) automated export procedures for seamless data downloads to common statistical packages; and (4) procedures for data integration and interoperability with external sources.

### Interim assessment strategy

**Fig. 2:**
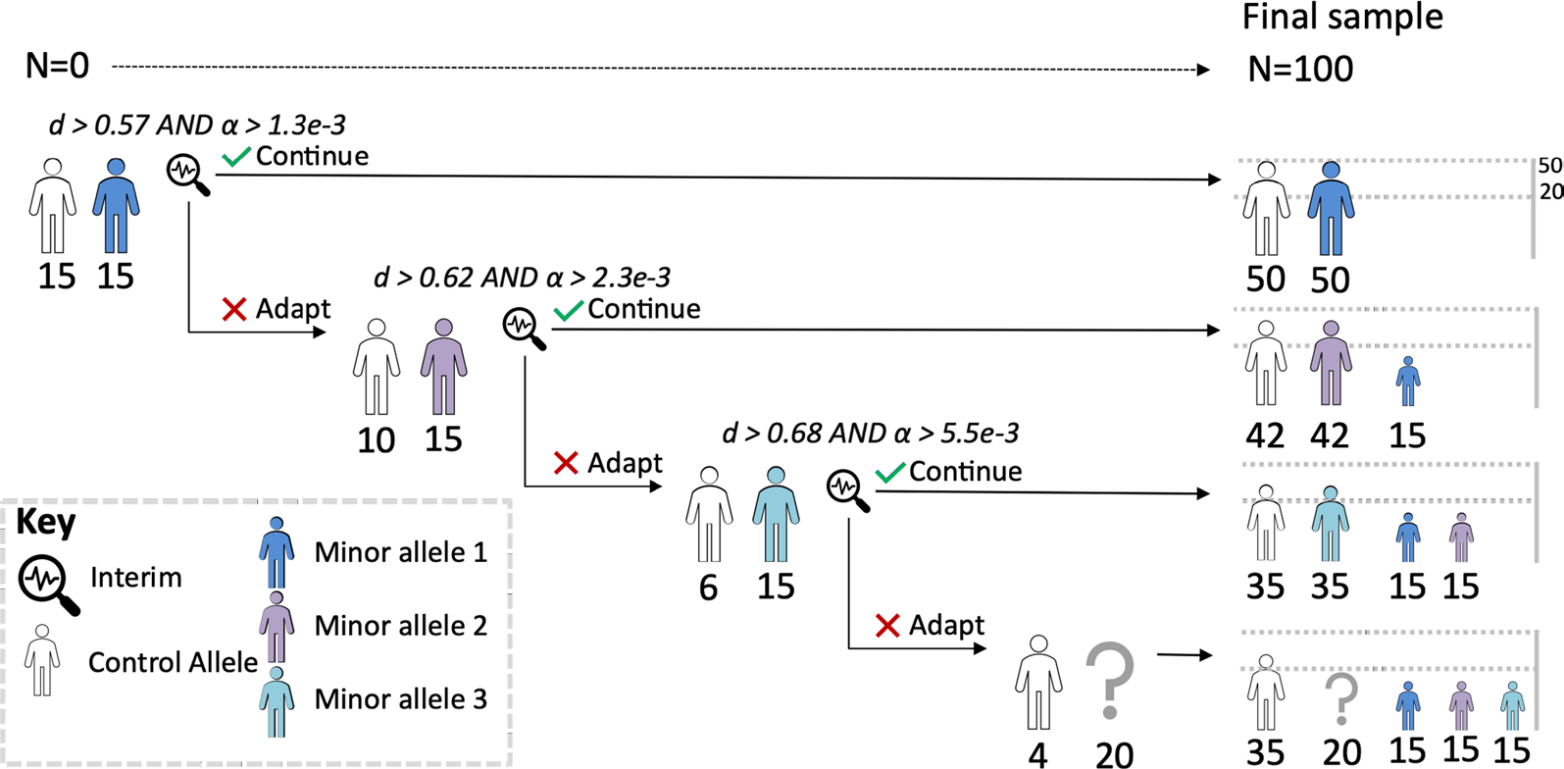
Schematic of study design showing the 4 potential outcomes of the adaptive design. The trial progresses from left to right until the full sample of 100 is recruited. Each allele cohort is noted with uniquely coloured human icon with the numbers recruited in that phase noted underneath. Interim assesments are marked with a magnifying glass with the effect size ‘d’ criteria to continue the trial noted and alpha thresholds for subsequent t-test. Note that the trial will adapt due to small effect sizes and also if the hypothesis test passess due efficacy. The final “?” represents that this final cohort could be from any of the final cohorts based on assesment of other outcomes. For further details on the outcome criteria see Table 2

Interim assessments will be used to determine if the recruitment of the minor allele group should be changed given the likelihood of observing a detectable effect (80% power, ⍺ ≤ 0.05 using independent two tailed t-test) at the end of the study (see Fig. 2). To reduce the impact of performing interim assessments, the criteria for adapting the study are stated a priori and an O’Brien-Fleming alpha spending is used after each hypothesis test [42].

**Fig. 3:**
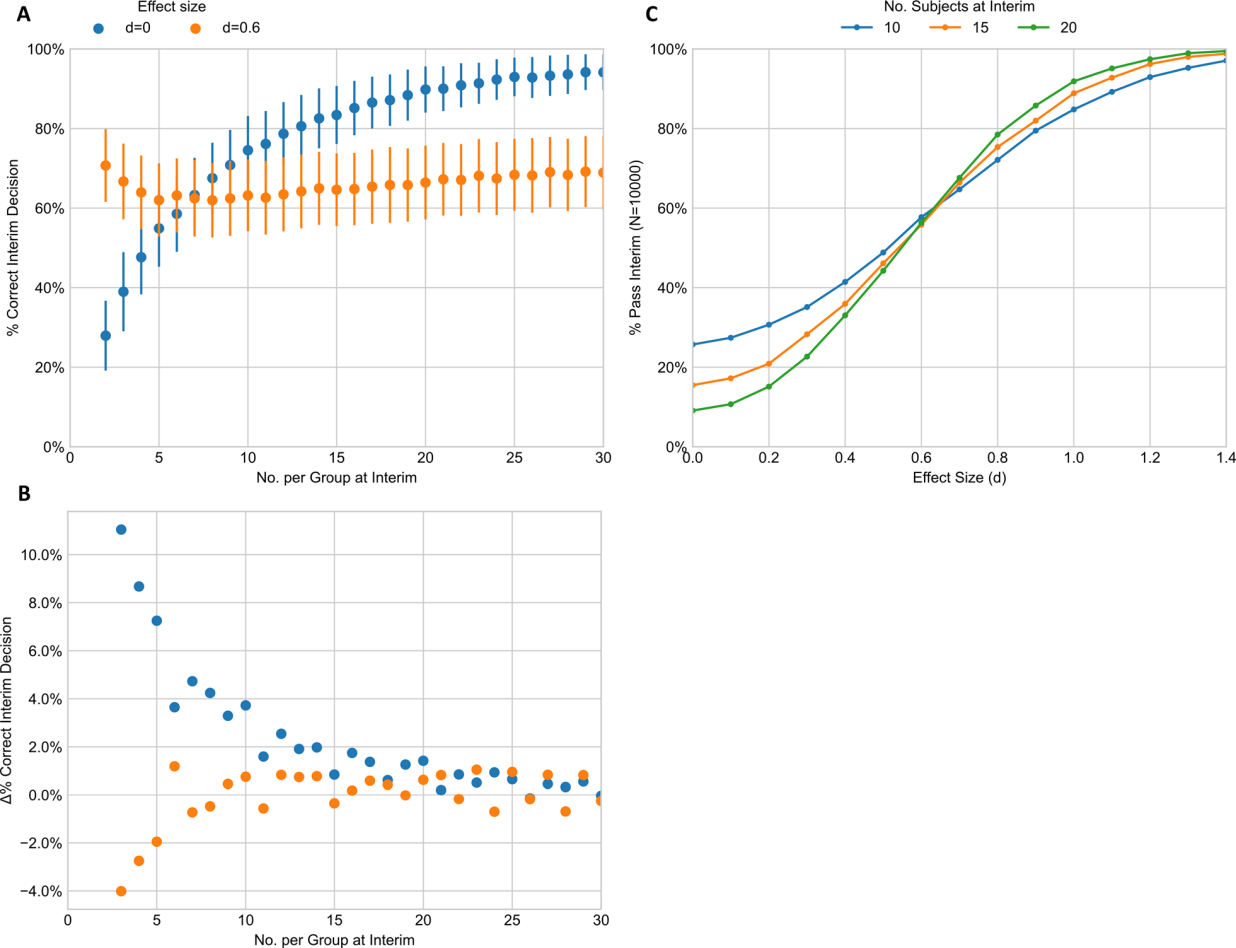
Simulations of the interim analysis. **A** The number of correct interim decisions—where an interim was stopped where there was no significant finding or continued for effect sizes of 0 and 0.6. Error bars indicate 95% confidence intervals generated by bootstrapping the simulation results sampling 100 results 1000 times. **B** The change in mean % correct interim decisions as more subjects are added to interim. The interim number 15 was chosen as the relative benefit of adding more decreases above this point. **C** The effect of number of subjects on overall interim pass rate. This figure displays the % of interims that would go on to recruit a full study from populations of fixed effect sizes

Individuals homozygous for the minor allele(s) and individuals from the control allele group will be recruited until at least 15 members have been recruited to both groups (as determined by simulations see Fig. 3). At this point, an interim analysis will be performed:

1. If the interim analysis predicts >  = 80% statistical power using an estimated effect size then a hypothesis test will be performed using O’Brien-Fleming alpha-spending criteria:
  If the null hypothesis cannot be rejected at this point then recruitment of this minor allele group along with the control group will continue to the end of the planned cohort.
  Else if the null hypothesis can be rejected then this minor allele group will stop. The study will continue with recruitment of the next planned minor allele group.
2. If the interim analysis predicts < 80% statistical power then recruitment of this minor allele group will stop and individuals from next minor allele group will be recruited. Recruitment will again be continued up to the pre-specified interim analysis (repeat analysis step (1))

This process will be repeated iteratively until the full cohort has been assessed (N = 100) as directed by the recruitment plan (see Fig. 2).

The interim and final analyses will use data from the Control group as the comparator therefore, to maintain equally sized groups at the end of the study and to maintain study group masking, the rate of recruitment into the Control group will reduce after each adaptation in minor allele group recruitment. The changes in recruitment strategy will be directed by the interim analysis committee and implemented by an independent group within the ALSPAC participant recruitment team.

In the event of all minor alleles being underpowered for differences in heat pain threshold, as determined by the interim assessments, additional endpoints derived from the secondary measures (QST and flare) will inform the final group recruitment (see Fig. 3 and Table 2 which illustrate the adaptive design and its effects upon recruitment).

**Table 2:** Interim analyses

Interim analysis #	Futility effect size	Efficacy cut-off alpha	Effect size req. for statistical eff (ΔHPT)
1	0.57	0.0013	1.63 (Δ4.1 °C)
2	0.62	0.0023	1.52 (Δ3.8 °C)
3	0.68	0.0055	1.42 (Δ3.5 °C)

Each row indicates the study state at subsequent interims. Effect sizes are displayed as Cohen’s d $\frac{{\left( {\mu_{minor} - \mu_{major} } \right)}}{\sigma }$ where µ is the mean and σ is the standard deviation. The futility effect size cut-off is the minimum effect size required to continue the group in the study. The futility effect size is calculated as the smallest effect size observable at the final analysis at 80% probability given an alpha cut-off of 0.05. The efficacy cut-off alpha is the alpha criteria for the interim analysis to determine efficacy. The efficacy cut-off alpha is calculated using an O’Brien-Fleming alpha spending through the gsdesign R package. With subsequent interims the alpha threshold is relaxed as the proportion of information known at interim increases due to the fixed sample size of 15. The effect size required for statistical efficacy (HPT) indicates the minimum effect size (cohen’s d) likely to be observed at 80% power at interim with the corresponding changes in HPT (Δ) indicated assuming σ = 2.5. HPT, heat pain threshold

We will follow the applicable Food and Drug Administration (FDA) guidelines for adaptive trial design [43]. To maintain the integrity of the sampling frame through this experiment, the analysis script is stated a priori and researchers involved in the data collection will not be aware of the outcomes of any interim analysis nor changes in the recruitment strategy; and therefore remain blind to genotype throughout.

### Simulation

To determine the timing of the interim analysis, simulations of the study design were performed. Hypothetical results were drawn equal to the number of participants (50) from separate normal distributions of fixed effect sizes ranging from *d* = 0 (null) to *d* = 1.6 were subject to an emulated interim analysis after a varying number of participants had been ‘recruited’. A t-test was performed comparing the two sets and considered successful where a significance level α <  = 0.05. This was repeated 10,000 times for each combination to evaluate the effect of participant numbers on the number of trials that would either have: (1) Been prematurely halted, where an effect would have been observed had the trial completed (False Negative, type 2 error); or (2) Been incorrectly continued, where an effect was too small to be observed with confidence at the end of the study (False Positive, type 1 error).

### Data analysis

The hypothesis test at interim and final analysis will be performed using a two-sided independent t-test. The type-1 error is controlled using the O'Brien-Fleming alpha spending method for the primary outcome measure, the heat pain threshold. Other QST variables will only be analysed at the final analysis. All other comparisons between available groups will be treated as exploratory. The genotype of the individuals will also be considered to qualify the association of SNP to any observed effect.

### Data access statement

Data collected as part of this study will be available on request to the ALSPAC executive committee (alspac-exec@bristol.ac.uk). The ALSPAC data management plan (available here: http://www.bristol.ac.uk/alspac/researchers/data-access/) describes in detail the policy regarding data sharing, which is through a system of managed open access. Code used both in the work presented herein and in the statistical analysis itself will be made available from the corresponding author on reasonable request.

## Discussion

This study will utilise detailed sensory testing within a recall-by-genotype framework [11] to assess variation in pain sensitivity in healthy young adults due to commonly occurring SNPs in *TRPA1*. Importantly, this study advances the RbG approach via a novel pairing with an adaptive design using interim assessments. Our primary rationale for this approach is for deep phenotypic screening of minor allele carriers where it is difficult to estimate the effect size. By performing interim assessment our approach reduces futile assessment of participants when an effect is unlikely to be observed given a predetermined sample size and reduces excessive assessment of participants where there is a large effect.

### Comparison with standard designs

In this study we focused on the heat pain threshold (HPT) of QST as TRPA1 has been implicated in detection of thermal stimuli. The number of participants to recruit for a RbG study is typically calculated from the expected effect size of the variant, however, there is little reliable data upon which to base an effect size calculation for these *TRPA1* SNPs. When an effect size is unknown, an alternative approach is to use estimates of the minimum clinically relevant change. In a reference population, the HPT is 42 ± 2.5 °C (mean ± SD) [44] and we consider a 1.5 °C (*d* = 0.6) change in HPT as being a minimum clinically relevant change (Baron, Maier [45]). In a classic design the number of individuals required for the study can be calculated given a desired probability of observing the effect size at a defined error cut-off. In a standard RbG study design investigating a single minor allele 45 subjects (90 total) would be required in the minor and major allele groups with 80% likelihood of observing an effect at alpha <  = 0.05.

### Efficacy

The estimated effect size is commonly used to determine the number of participants to recruit for a study, however if the estimated mean effect size is lower than the actual mean effect within the sampled population then more participants will be recruited than is required to observe the effect. If we consider where we observe a large 4 °C change in heat pain threshold, a recall by genotype would have an 80% chance of observing this effect after the first seven participants per group (given our expected standard deviation 2.5 °C), however the full cohort would still have been recruited. In our study, there is a 77.6% chance of observing a 4 °C change at the first interim with fifteen participants per study arm resulting in less assessment of individuals with a hypersensitivity to noxious stimuli.

### Futility

Conversely, there is a chance that the actual mean effect within the sampled population is considerably smaller than the estimated mean effect size. In the classic design this will only be apparent once the full cohort is assessed. Using this adaptive design it is likely that the recruitment of the allele would be stopped at an interim assessment after only 30 participants are recruited. As such, our design reduces the burden on the experimenter and the participants when an effect is unlikely to be observed.

In many studies the sample size is restricted due to practical and financial concerns which can result in the study being of insufficient power to observe clinically relevant changes. In this study we have been resourced to recruit a total cohort of 100 participants where the adaptive design can screen up to 3 cohorts and is powered to detect changes of 1.43 °C (d = 0.57), 1.55 °C (d = 0.62) and 1.7 °C (d = 0.68) at each interim respectively. An analogous, “classical” RbG with multi-armed assessment with 25 participants per group would be powered to detect a difference of 2.03 °C (*d* = 0.81 α <  = 0.05) in the heat pain threshold.

### Statistical considerations

Unblinded assessment of effect size at interim has the potential to impact the final statistical analysis. For example, if an interim analysis performs a hypothesis test to reject the null hypothesis then it introduces a testing multiplicity which will increase the likelihood of type 1 errors (false positives) in the final analysis. A common approach to take account of the interim assessment is to split the alpha criteria across the additional interims, referred to as alpha spending. In its simplest form the critical value threshold is divided equally amongst the interims including the final analysis [46]. For example, the addition of an interim analysis with a desired final alpha of 0.05 results in an adjusted alpha threshold of 0.025 at the interim and final analysis stage giving an equal weight to both analyses. More commonly, alpha spending strategies which consider the available information at the time of interim assessment are employed such as the O’Brien–Fleming method, which divides the alpha threshold by the number recruited at interim relative to the total number of participants [42]. In the case where a single interim is performed after half of the participants are recruited the effect is the same as the Pocock adjustment. However, where the interim occurs with less than half, a larger effect size is required to stop for efficacy than at the end of the study which accounts for the lower confidence associated with the fewer participants earlier in the study. We opted for the O’Brien-Fleming approach as we desired to perform interim at an early timepoint to allow screening of more alleles; we expect small effect sizes and therefore will only stop for efficacy at interim where the effect is very robust; and desire the interim to have minimal impact on the final analysis.

### Simulations

The optimal point (number of participants) at which to perform the interim analysis is determined by statistical and practical considerations with the objective of making effective use of the scarce and valuable resource of the available cohort, in our case ALSPAC. Ideally, at interim, the distributions of the data should be representative of the final distribution; if not it may lead to an incorrect decision to continue or halt the experiment. We undertook simulations of the study design to better estimate this optimal interim point. We examined a broad range of possible effect sizes, but chose to focus on the characteristics under the null effect (d = 0) and the minimal clinically relevant effect size (d = 0.6). Assessment of the modelling demonstrates that an interim analysis after 15 participants per group using a confidence threshold of 80% would adequately balance this risk, whilst still allowing multiple alleles to be tested. As can be seen from Fig. 3, with 15 participants per group, we can be more than 80% confident that we will correctly halt approaching 90% of “negative” trials balanced against the risk of incorrectly halting approximately 10% of “positive” trials. Further increasing the number at interim beyond 15 had little impact on the efficiency of the interim assessment. As our sample size is fixed by available resource and statistical power is determined from effect size and sample size, then here the effect size will determine the result of the interim assessment.

### Other adaptive designs

There are many varieties of adaptive design which could be applied to RbG studies. We chose to run sequential allele groups in our recruitment which was ordered on prior knowledge, allele frequency and confidence in the assocations. Where there is equal confidence in the alleles, an alternative adaptive design could recruit from all allele groups, and after interim adapt recruitment to a single group which creates a larger cohort to recruit from at the start of the study.

## Concluding remarks

The novel approach to a recall by genotype study presented herein will allow efficient assessment of the contribution of common SNPs to pain sensitivity and offers an alternative approach to understanding the polygenic contributions to this complex and heterogeneous phenotype. In addition, this approach prevents unnecessary testing of individuals where there is unlikely to be an effect on the phenotype of interest, which is an important ethical consideration. We suggest that this approaches’ ability to maximise both exploratory potential and resources can be applied across all RbG settings.

## Supplementary Information

**Additional file 1**. ALSPAC: Genotyping and imputation description.

## Data Availability

Data collected as part of this study will be available on request to the ALSPAC executive committee (alspac-exec@bristol.ac.uk). The ALSPAC data management plan (available here: http://www.bristol.ac.uk/alspac/researchers/data-access/) describes in detail the policy regarding data sharing, which is through a system of managed open access. Code used both in the work presented herein and in the statistical analysis itself will be made available from the corresponding author (james.p.dunham@bristol.ac.uk) on reasonable request.

## Abbreviations

AITC: Allyl isothiocyanate
ALSPAC: Avon longitudinal study of parents and children
CFA: Coal fly ash
DFNS: German research network on neuropathic pain (Deutsche Forschungsverbund Neuropathischer Schmerz)
FDA: Food and Drug Administration
FLPI: Full-field laser perfusion imaging
GWAS: Genome wide association study
HEK: Human embryonic kidney cells
HPT: Heat pain threshold
IMMPACT: Initiative on methods, measurement, and pain assessment in clinical trials
MAF: Minor allele frequency
QST: Quantitative sensory testing
RbG: Recall by genotype
SNPs: Single nucleotide polymorphisms
TRPA1: Transient receptor potential A1
