# Supplementary material for: Evaluating the association of TRPA1 gene polymorphisms with pain sensitivity: a protocol for an adaptive recall by genotype study
Source: BMC Med Genomics. 2022 Jan 12;15:9. doi: 10.1186/s12920-022-01156-5 (PMC8753821; doi:10.1186/s12920-022-01156-5)
Supplement: Supplementary file 1 — Additional file 1. ALSPAC: Genotyping and imputation description. [file 12920_2022_1156_MOESM1_ESM.docx]

**Supplementary Information**

**ALSPAC: Genotyping and imputation description**

Members of the original ALSPAC cohort (individuals born between 1990 and 1992) were genotyped using the Illumina HumanHap550 quad chip genotyping platforms by 23andme subcontracting the Wellcome Trust Sanger Institute, Cambridge, UK and the Laboratory Corporation of America, Burlington, NC, US. The resulting raw genome-wide data were subjected to standard quality control methods. Individuals were excluded on the basis of sex mismatches; minimal or excessive heterozygosity; disproportionate levels of individual missingness (>3%) and insufficient sample replication (identity by descent (IBD) < 0.8). Population stratification was assessed by multidimensional scaling analysis and compared with Hapmap II (release 22) European descent (CEU), Han Chinese, Japanese and Yoruba reference populations; all individuals with non-European ancestry were removed. SNPs with a minor allele frequency of less than 1%, a call rate of < 95% or evidence for violations of Hardy-Weinberg equilibrium (P < 5E-07) were removed. Cryptic relatedness was measured as proportion of identity by descent (IBD > 0.1). Related subjects that passed all other quality control thresholds were retained during subsequent phasing and imputation. 9,115 subjects and 500,527 SNPs passed these quality control filters.

Mothers of the original cohort were genotyped using the Illumina human660W-quad array at Centre National de Génotypage (CNG) and genotypes were called with Illumina GenomeStudio. PLINK (v1.07)(1) was used to carry out quality control measures on an initial set of 10,015 subjects and 557,124 directly genotyped SNPs. SNPs were removed if they displayed more than 5% missingness or a Hardy-Weinberg equilibrium P value of less than 1E-06. Additionally, SNPs with a minor allele frequency of less than 1% were removed. Samples were excluded if they displayed more than 5% missingness, had indeterminate X chromosome heterozygosity or extreme autosomal heterozygosity. Samples showing evidence of population stratification were identified by multidimensional scaling of genome-wide identity by state pairwise distances using the four HapMap populations (see above) as a reference, and then excluded. Cryptic relatedness was assessed using a IBD estimate of more than 0.125 which is expected to correspond to roughly 12.5% alleles shared IBD or a relatedness at the first cousin level. Related subjects that passed all other quality control thresholds were retained during subsequent phasing and imputation. 9,048 subjects and 526,688 SNPs passed these quality control filters.

477,482 SNP genotypes in common between the sample of mothers and sample of children (original cohort) were combined. SNPs with genotype missingness above 1% due to poor quality were removed (11,396 SNPs removed). A further 321 subjects were removed due to potential ID mismatches. This resulted in a dataset of 17,842 subjects containing 6,305 duos and 465,740 SNPs (112 were removed during liftover and 234 were out of Hardy-Weinberg equilibrium after combination). Haplotypes were estimated using ShapeIT (v2.r644) which utilises relatedness during phasing. Imputation of the target data was performed using IMPUTE V3 against the Haplotype Reference Consortium (r1.1) imputation panel(2, 3). This procedure gave imputed data for 17,842 mothers and children; subsequent consent withdrawals have left 17,825 individuals for study.

1. Purcell S, Neale B, Todd-Brown K, Thomas L, Ferreira MA, Bender D, et al. PLINK: a tool set for whole-genome association and population-based linkage analyses. Am J Hum Genet. 2007;81(3):559-75.

2. Howie BN, Donnelly P, Marchini J. A Flexible and Accurate Genotype Imputation Method for the Next Generation of Genome-Wide Association Studies. PLOS Genetics. 2009;5(6):e1000529.

3. Howie B, Marchini J, Stephens M. Genotype imputation with thousands of genomes. G3 (Bethesda). 2011;1(6):457-70.
